# Supplementary material for: Bioactive PKS–NRPS Alkaloids from the Plant-Derived Endophytic Fungus Xylaria arbuscula
Source: Molecules. 2021 Dec 27;27(1):136. doi: 10.3390/molecules27010136 (PMC8746755; doi:10.3390/molecules27010136)

Supporting Information

Bioactive PKS-NRPS alkaloids from a plant-derived endophytic Fungus *Xylaria arbuscula*

Ya Wang[1,†], Sinan Zhao[1,2,†], Tao Guo[3], Li Li[4], Tantan Li, Anqi Wang[2], Dandan Zhang[2], Yanlei Wang[2], and Yi Sun[2*]

[1] School of life science and engineering, Lanzhou University of Technology, Lanzhou 730050, China;

[2] Institute of Chinese Materia Medica, China Academy of Chinese Medical Sciences, Beijing100010, China;

[3] Henan University of Chinese Medicine, Zhengzhou 450046, China;

[4] Institute of Materia Medica, Chinese Academy of Medical Sciences & Peking Union Medical College, Beijing100010, China.

*Correspondence: Yi Sun, ysun@icmm.ac.cn.

† These authors contributed equally to this work.

Contents	Page
The ITS sequences data of *Xylaria arbuscula*	2
Figure S1: ^{1}H NMR (600 MHz, DMSO-d_6) spectrum of **1**	3
Figure S2: ^{13}C NMR (150 MHz, DMSO-d_6) spectrum of **1**	3
Figure S3: HSQC spectrum of **1**	4
Figure S4: HMBC spectrum of **1**	4
Figure S5: ^{1}H-^{1}H COSY spectrum of **1**	5
Figure S6: TOESY spectrum of **1**	5
Figure S7: NOESY spectrum of **1**	6
Figure S8: 1D NOE spectrum of **1**	6
Figure S9: HRESI-MS spectrum of **1**	7
Figure S10: ^{1}H NMR (600 MHz, DMSO-d_6) spectrum of **2**	8
Figure S11: ^{13}C NMR (150 MHz, DMSO-d_6) spectrum of **2**	8
Figure S12: ^{1}H NMR (600 MHz, DMSO-d_6) spectrum of **3**	9
Figure S13: ^{13}C NMR (150 MHz, DMSO-d_6) spectrum of **3**	9
Figure S14: ^{1}H NMR (600 MHz, DMSO-d_6) spectrum of **4**	10
Figure S15: ^{13}C NMR (150 MHz, DMSO-d_6) spectrum of **4**	10
Figure S16: ^{1}H NMR (600 MHz, DMSO-d_6) spectrum of **5**	11
Figure S17: ^{13}C NMR (150 MHz, DMSO-d_6) spectrum of **5**	11
Figure S18: ^{1}H NMR (600 MHz, DMSO-d_6) spectrum of **6**	12
Figure S19: ^{13}C NMR (150 MHz, DMSO-d_6) spectrum of **6**	12
Figure S20: ^{1}H NMR (600 MHz, DMSO-d_6) spectrum of **7**	13
Figure S21: ^{13}C NMR (150 MHz, DMSO-d_6) spectrum of **7**	13
Figure S22: ^{1}H NMR (600 MHz, DMSO-d_6) spectrum of **8**	14
Figure S23: ^{13}C NMR (150 MHz, DMSO-d_6) spectrum of **8**	14
Figure S24: ^{1}H NMR (600 MHz, DMSO-d_6) spectrum of **9**	15
Figure S25: ^{13}C NMR (150 MHz, DMSO-d_6) spectrum of **9**	15
Figure S26: ^{1}H NMR (600 MHz, DMSO-d_6) spectrum of **10**	16
Figure S27: ^{13}C NMR (150 MHz, DMSO-d_6) spectrum of **10**	16
Figure S28: ^{1}H NMR (600 MHz, DMSO-d_6) spectrum of **11**	17
Figure S29: ^{13}C NMR (150 MHz, DMSO-d_6) spectrum of **11**	17

The ITS sequences data of *Xylaria arbuscula*

CCGTAGGTGAACCTGCGGAGGGATCATTAAAGAGTTAAAACAACTCCTAAACCCATGTGAACCT
ACCTTTGTTGCCTCGGCAGGTCTGCAACTTACCCCGAGGGGACCTACCCTGTAGGGACCTTACC
CGGTAGTTGCGGGCATAACCTGCCGGTGGTCTACTAAACTCTGTTTACTATGTTATTCTGAATAA
TATAACTAAATAAGTTAAAACTTTCAACAACGGATCTCTTGGTTCTGGCATCGATGAAGAACGCA
GCGAAATGCGATAAGTAATGTGAATTGCAGAATTCAGTGAATCATCGAATCTTTGAACGCACAT
TGCGCCCATTAGTATTCTAGTGGGCATGCCTGTTCGAGCGTCATTTCAACCCTTAAGCCCTGTTG
CTTAGCGTTGGGAGCCTACAGATACCCTCTGTAGTTCCTTAAAGTTAGTGGCGGAGTCGGTTTCA
CACTCTAGACGTAGTAAATTTTATCTCGCCTATAGATGAGCCGGTCCCTTGCCGTAAAACCCCCT
AATTTCTAAAAGTTGACCTCGGATCAGGTAGGAATACCCGCTGAACTTAAGCAT

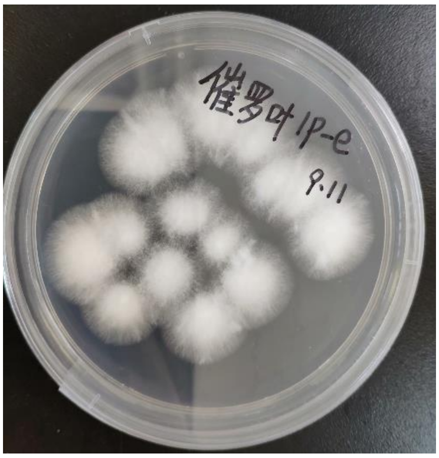

Figure S1: ^{1}H NMR (600 MHz, DMSO-d_6) spectrum of (**1**)

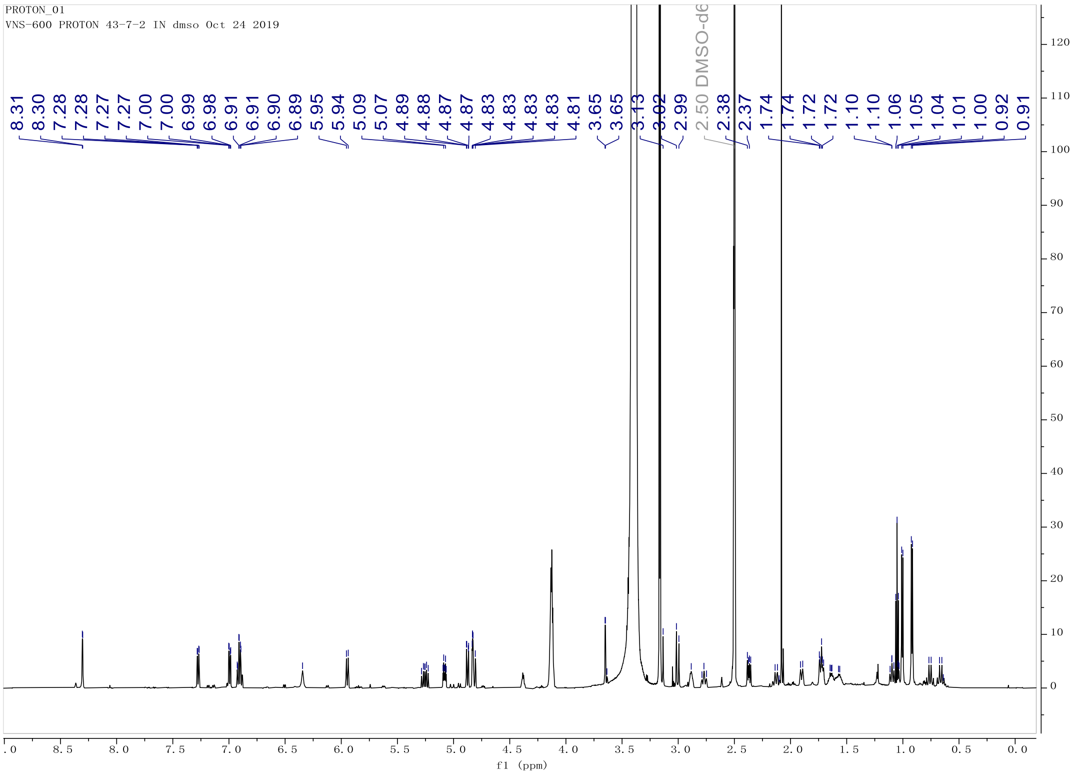

Figure S2: ^{13}C NMR (150 MHz, DMSO-d_6) spectrum of (**1**)

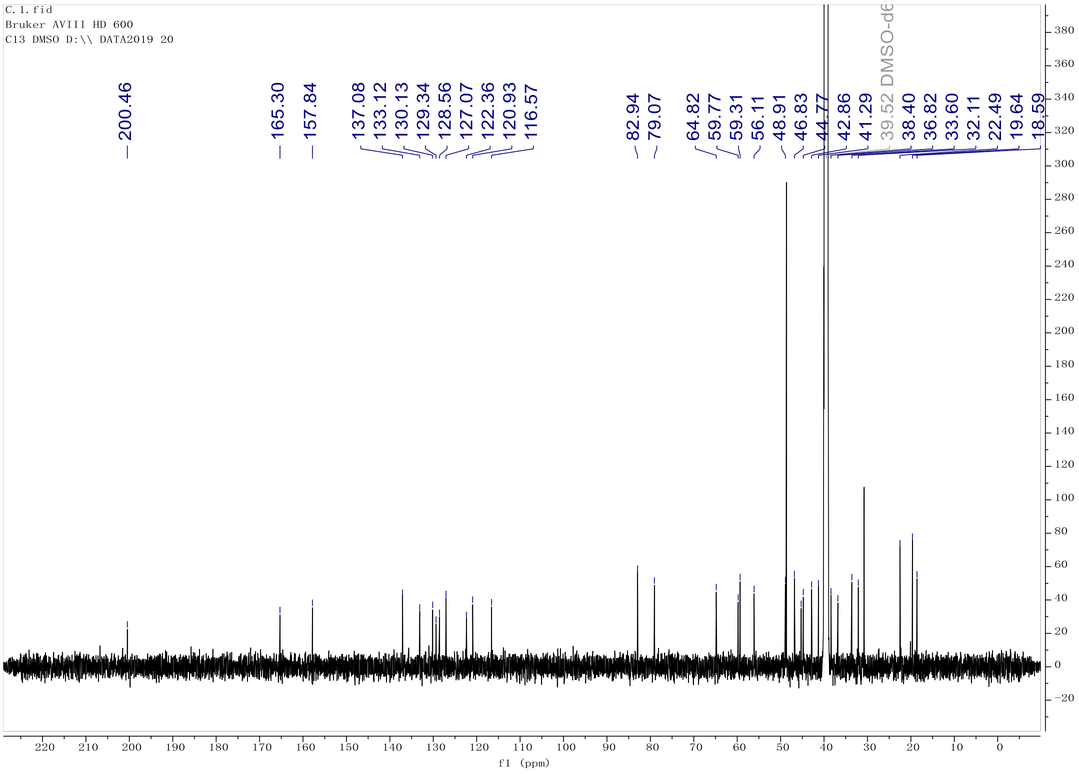

Figure S3: HSQC spectrum of (**1**)

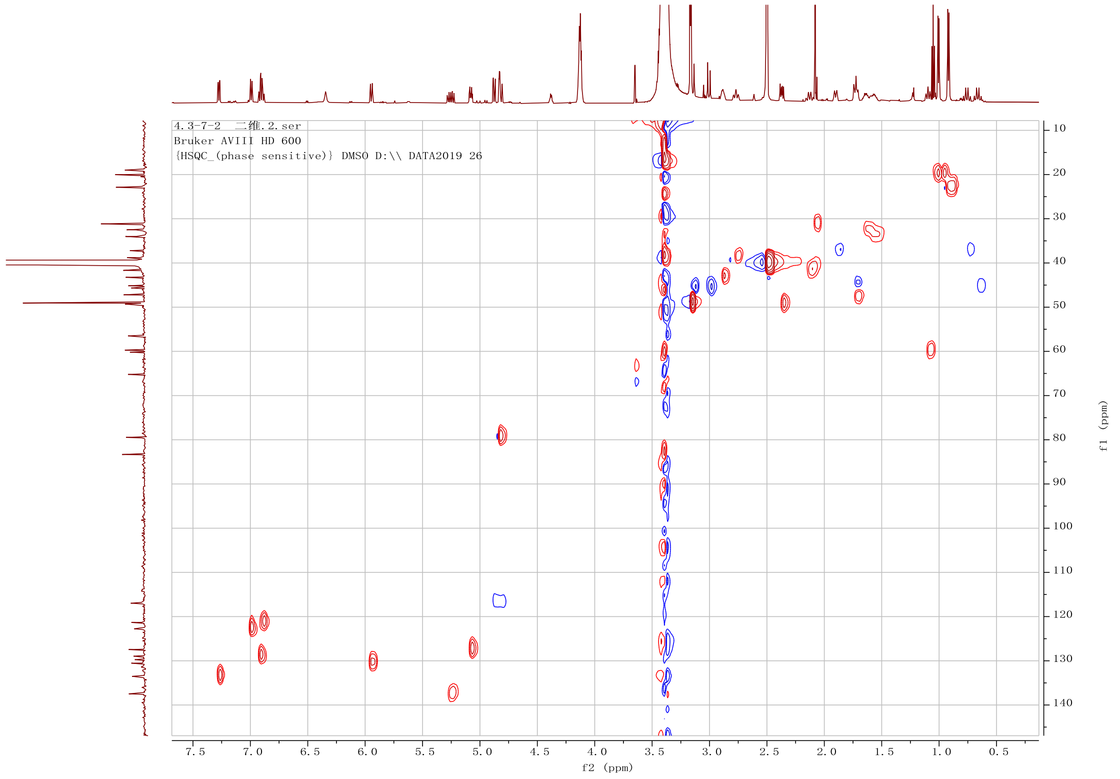

Figure S4: HMBC spectrum of (**1**)

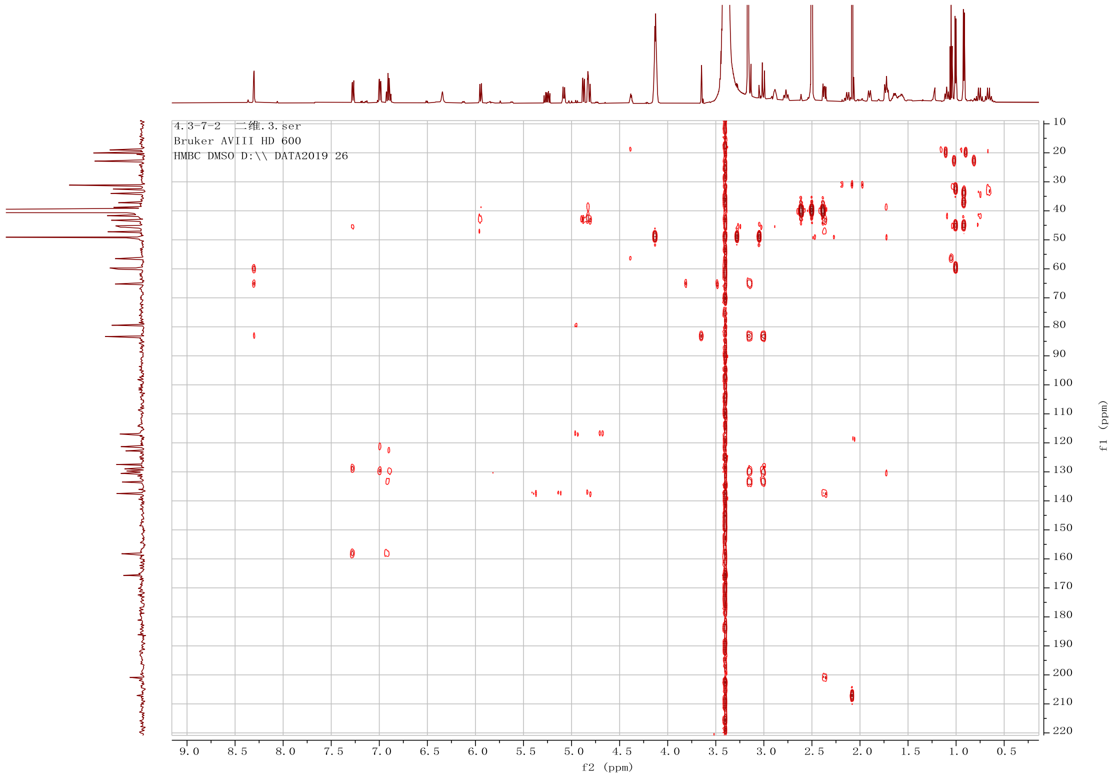

Figure S5: ^{1}H-^{1}H COSY spectrum of (**1**)

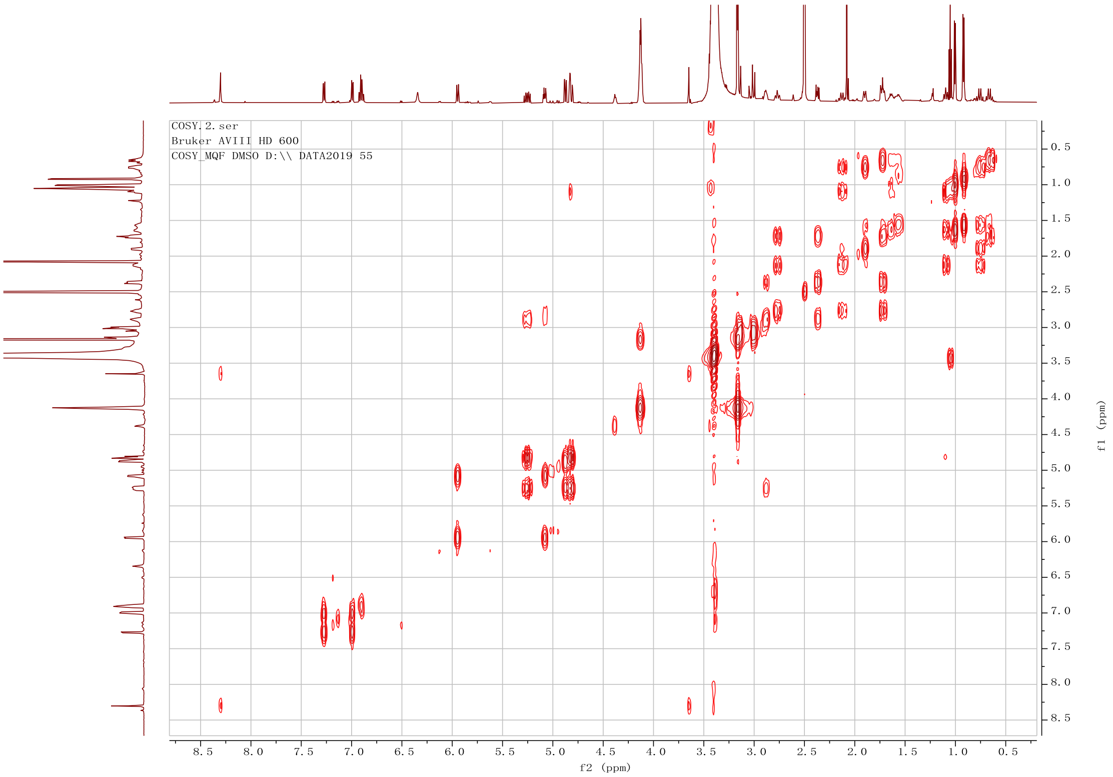

Figure S6: TCOSY spectrum of (**1**)

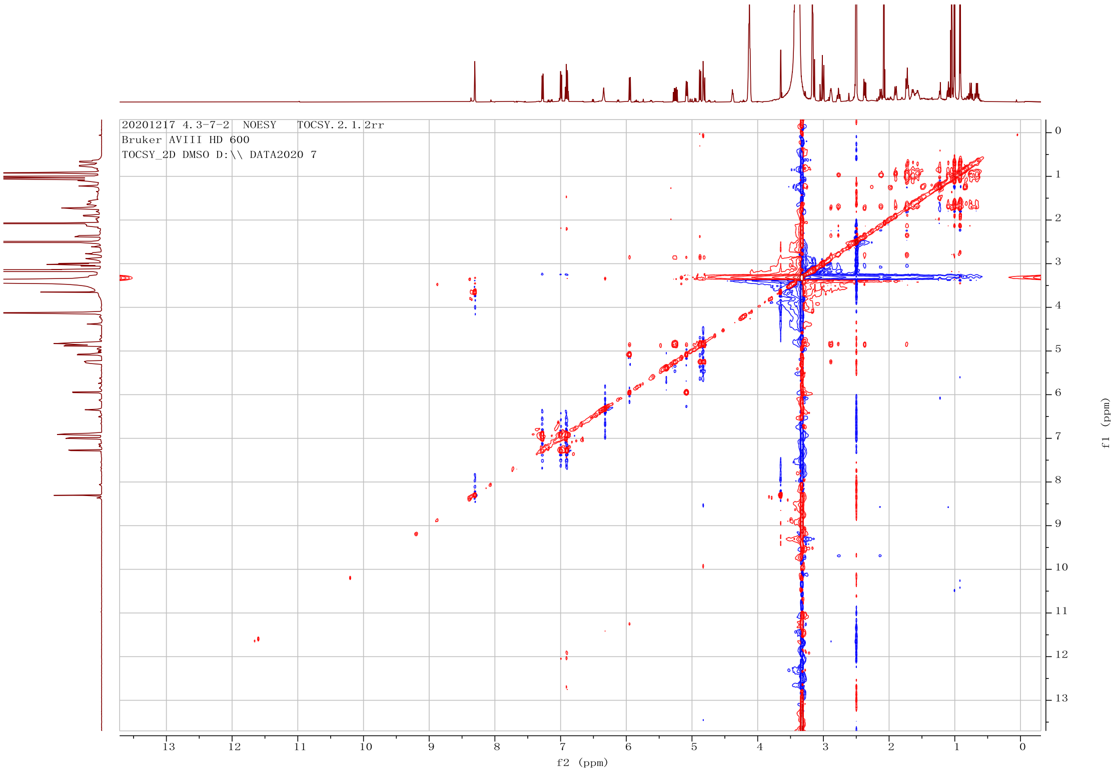

Figure S7: NOESY spectrum of (**1**)

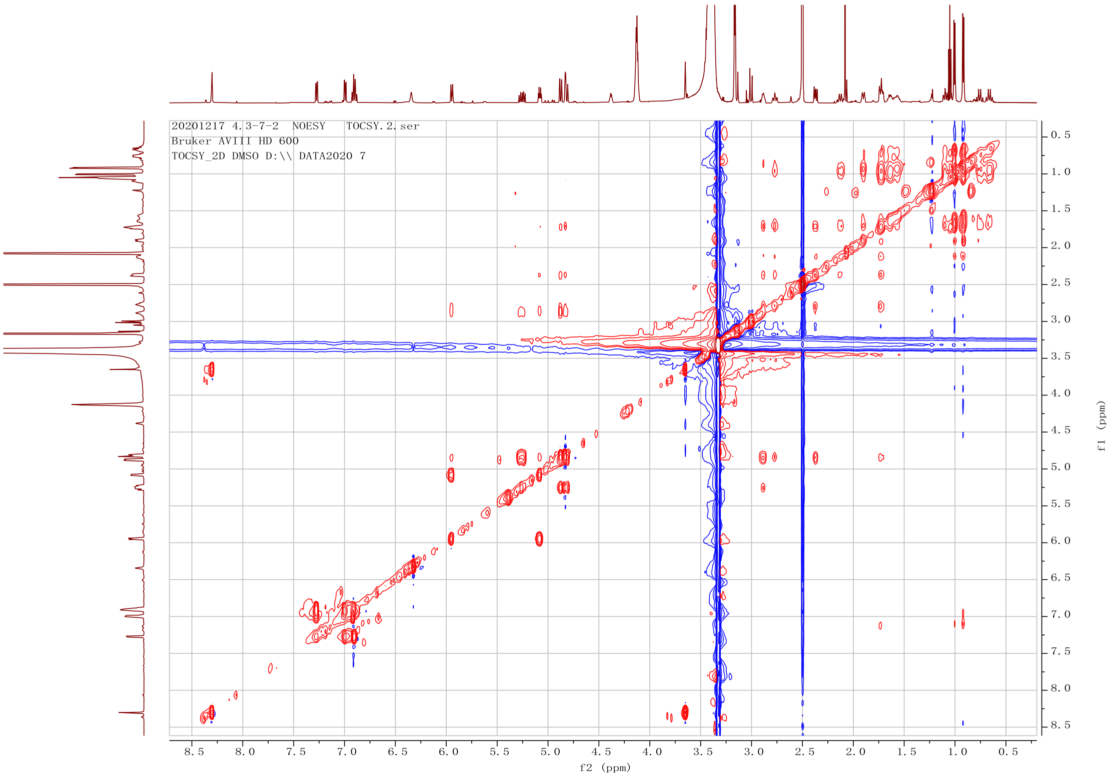

Figure S8: 1D NOE spectrum of **1**

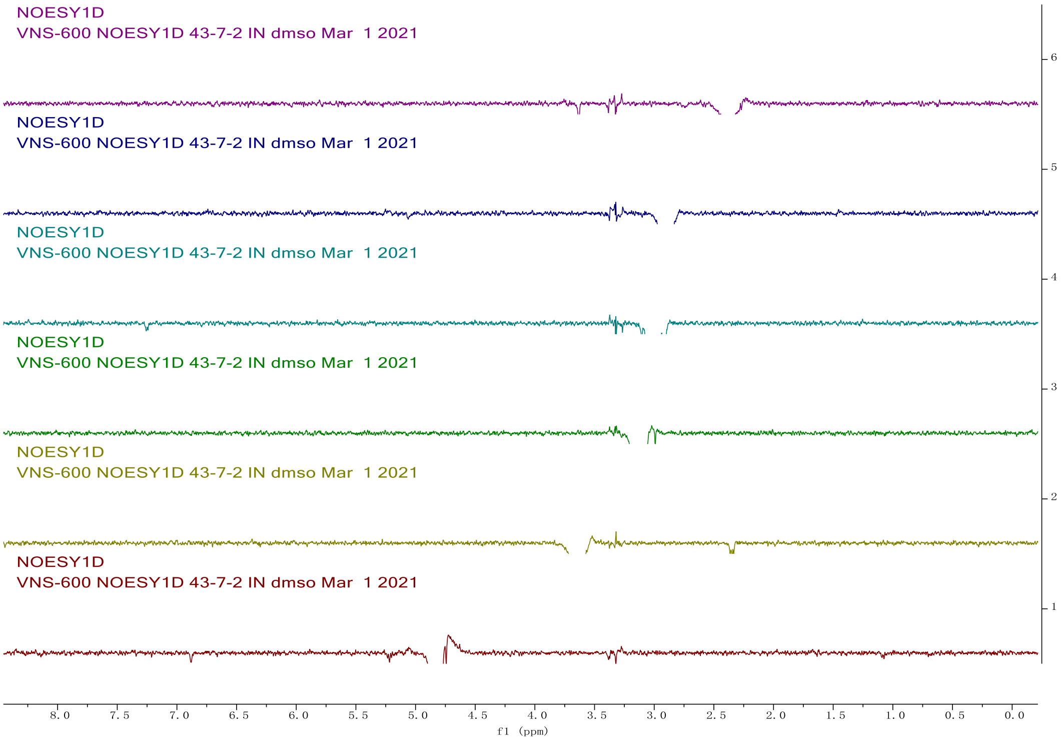

Figure S9: HRESI-MS spectrum of **1**

HRMS (ESI-TOF) *m/z*: $[M+H]^+$ Calcd for $C_{29}H_{33}NO_5$ 476.24368 ; Found 476.24315, Δ1.1 ppm.

Composition	i-FIT Confidence (%)	m/z RMS (PPM)	Intensity RMS (%)	Predicted m/z	m/z error (PPM)	m/z error (mDa)	DBE
C29H33NO5	88.367590	1.115058	3.125758	476.243150	1.116026	0.530376	14.000000

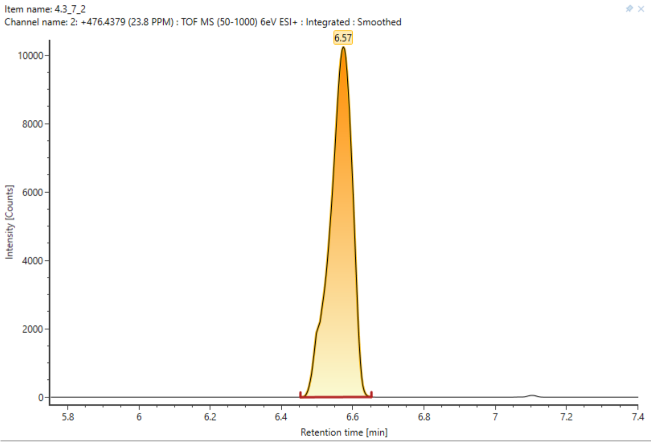

Figure S10: ^{1}H NMR (600 MHz, DMSO-d_6) spectrum of (**2**)

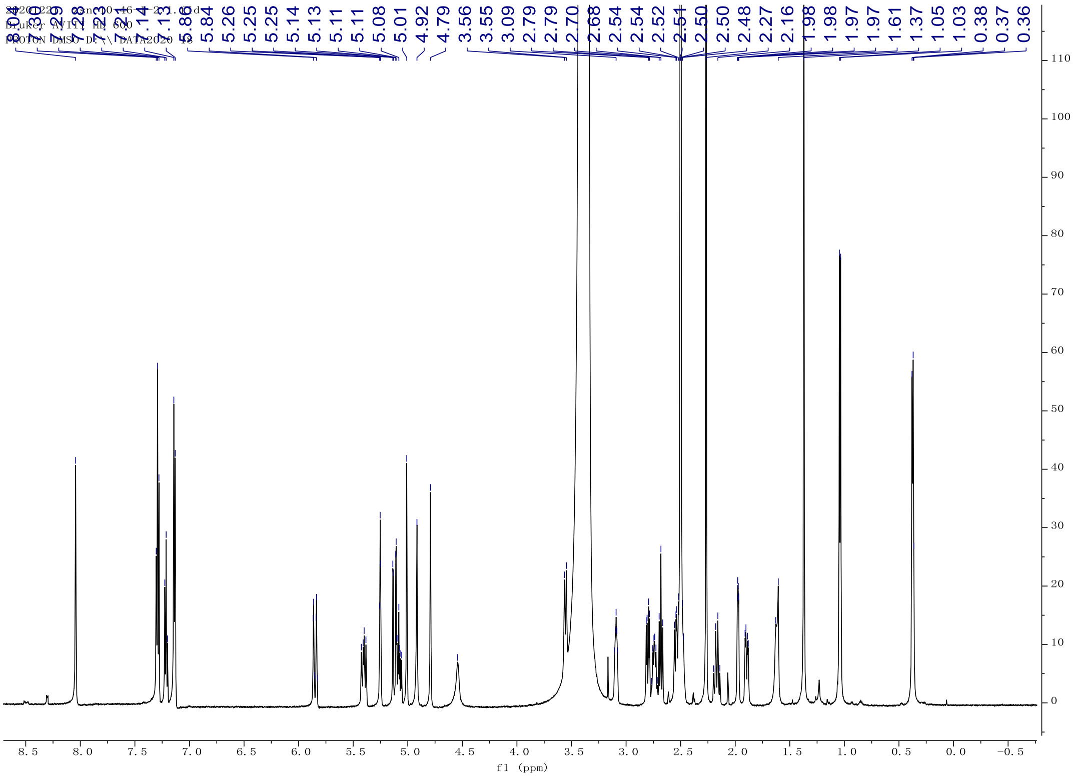

Figure S11: ^{13}C NMR (150 MHz, DMSO-d_6) spectrum of (**2**)

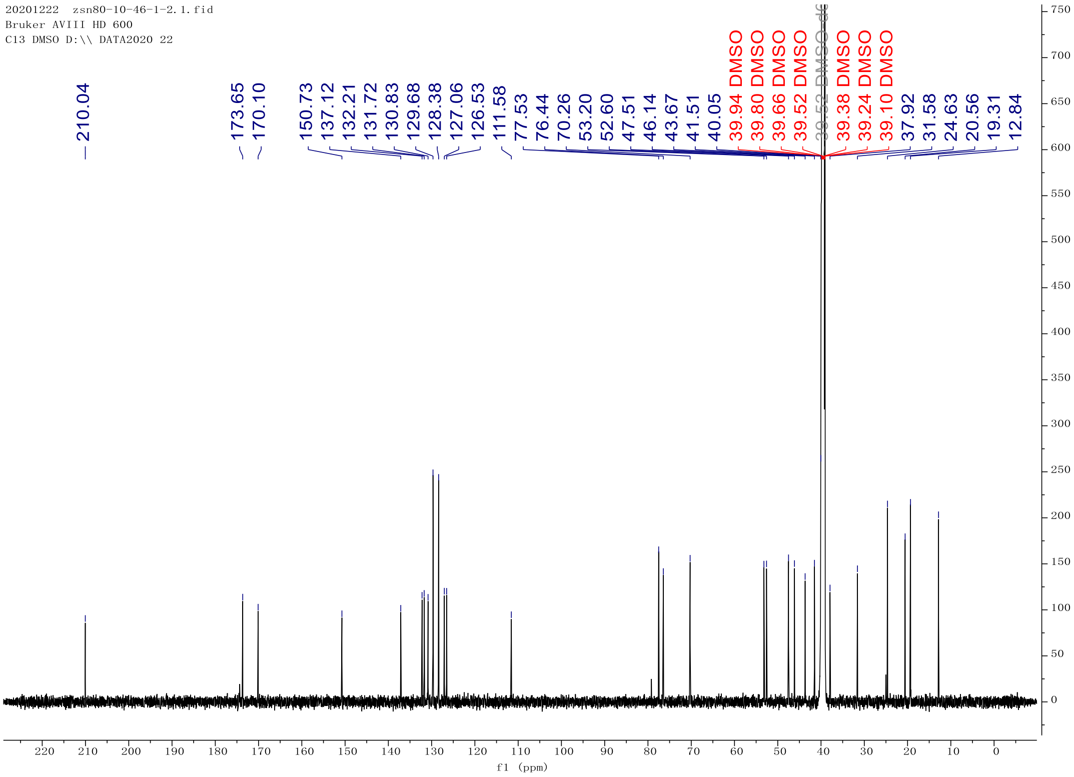

Figure S12: ^{1}H NMR (600 MHz, DMSO-d_6) spectrum of (**3**)

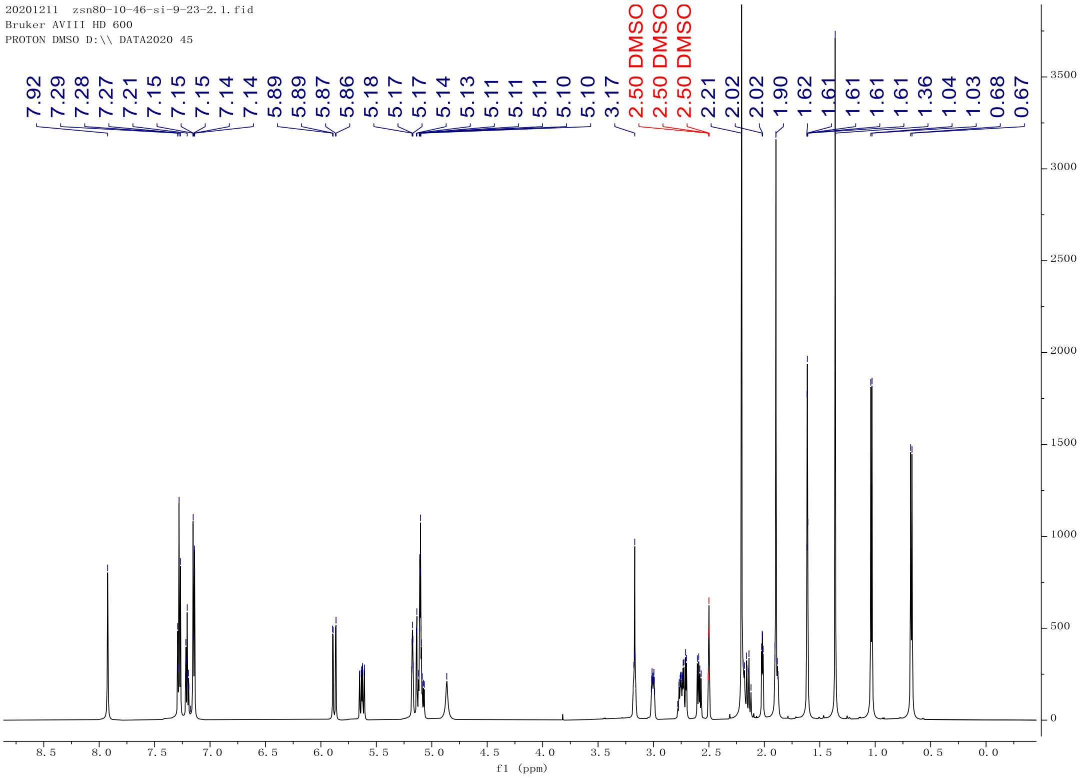

Figure S13: ^{13}C NMR (150 MHz, DMSO-d_6) spectrum of (**3**)

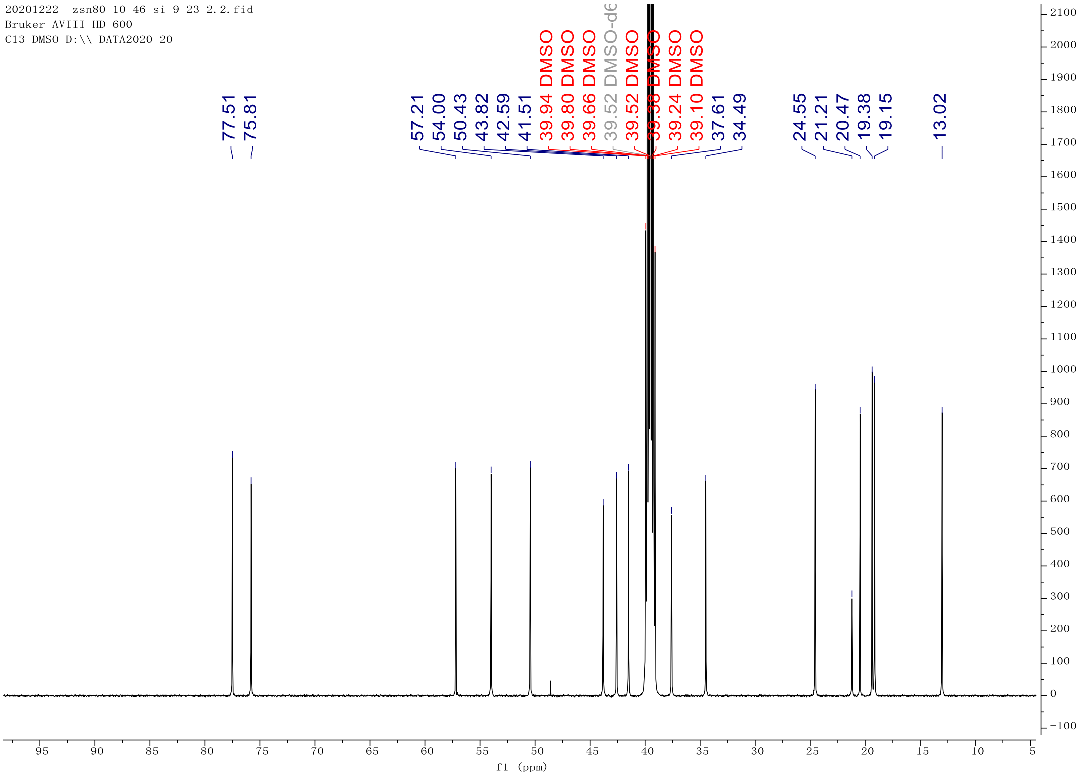

Figure S14: ^{1}H NMR (600 MHz, DMSO-d_6) spectrum of (**4**)

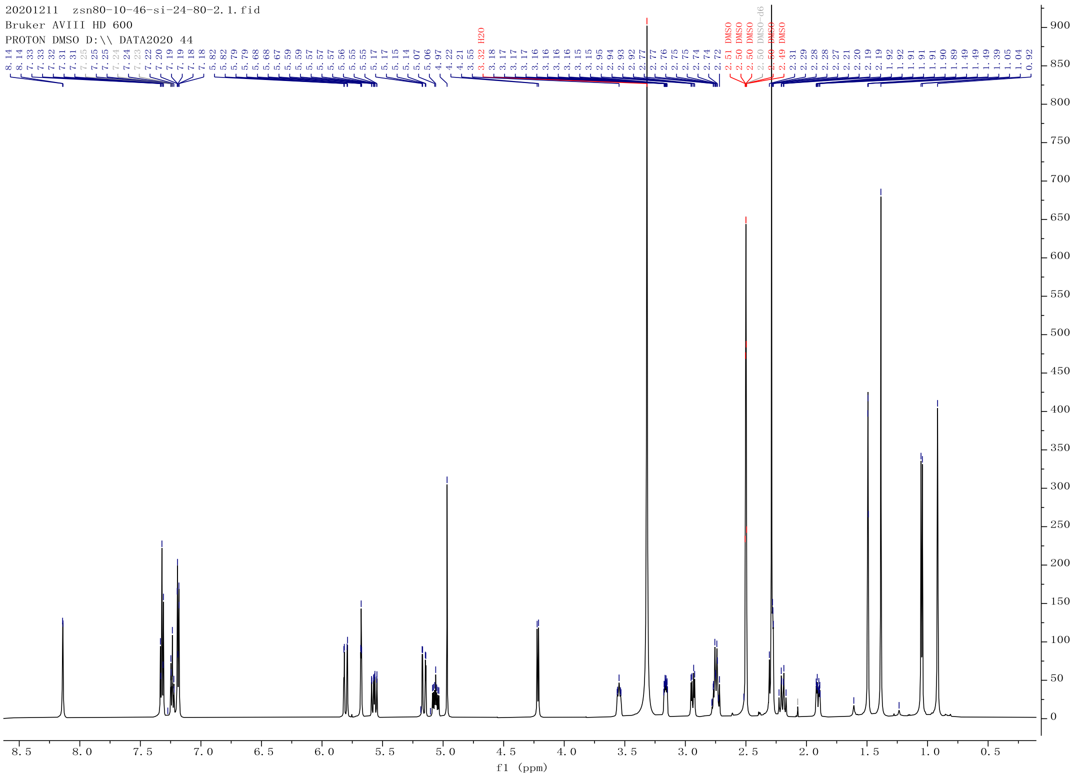

Figure S15: ^{13}C NMR (150 MHz, DMSO-d_6) spectrum of (**4**)

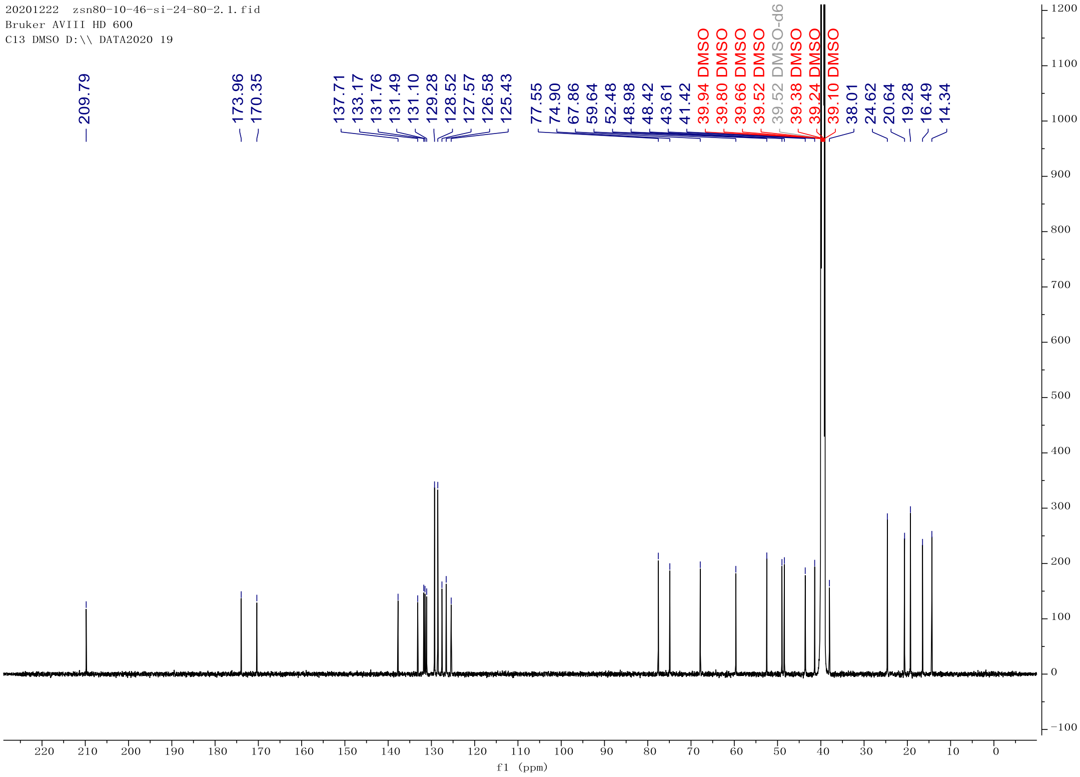

Figure S16: ^{1}H NMR (600 MHz, DMSO-d_6) spectrum of (**5**)

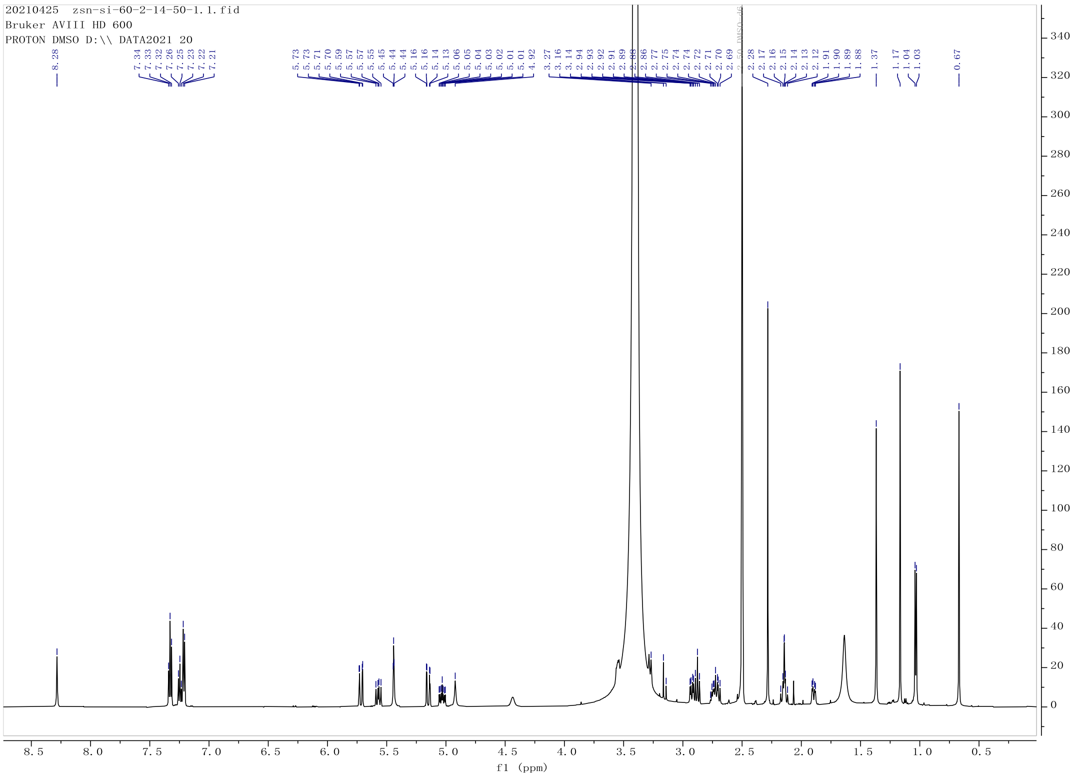

Figure S17: ^{13}C NMR (150 MHz, DMSO-d_6) spectrum of (**5**)

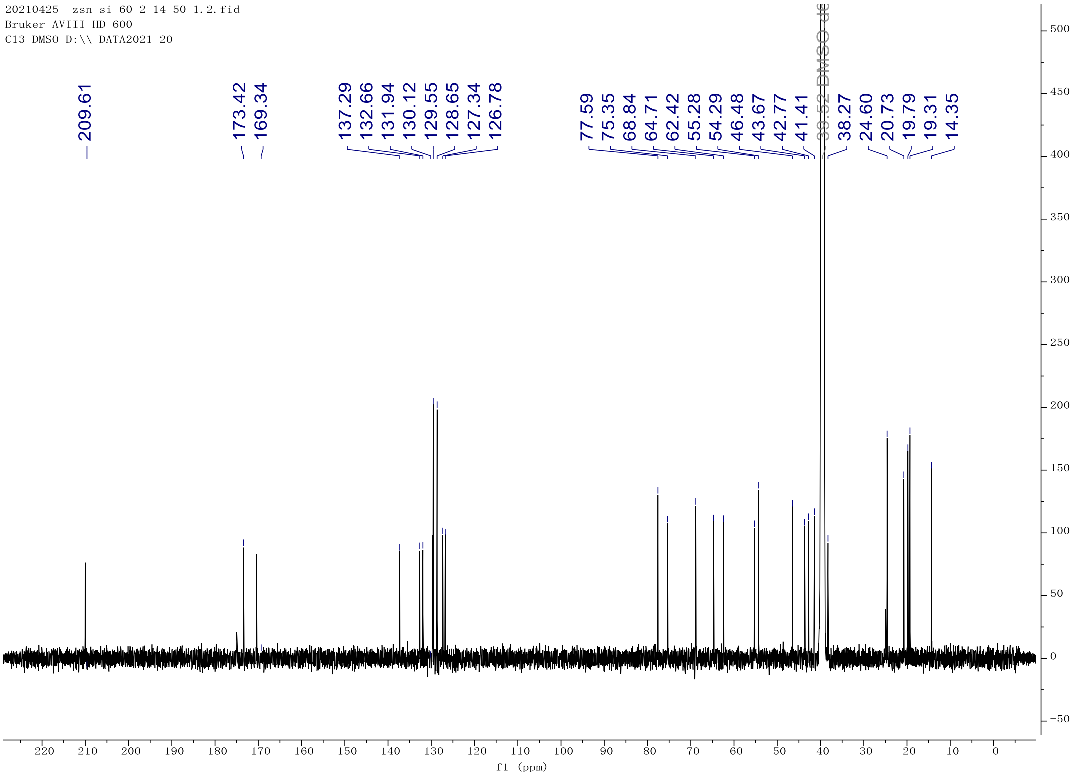

Figure S18: ^{1}H NMR (600 MHz, DMSO-d_6) spectrum of (**6**)

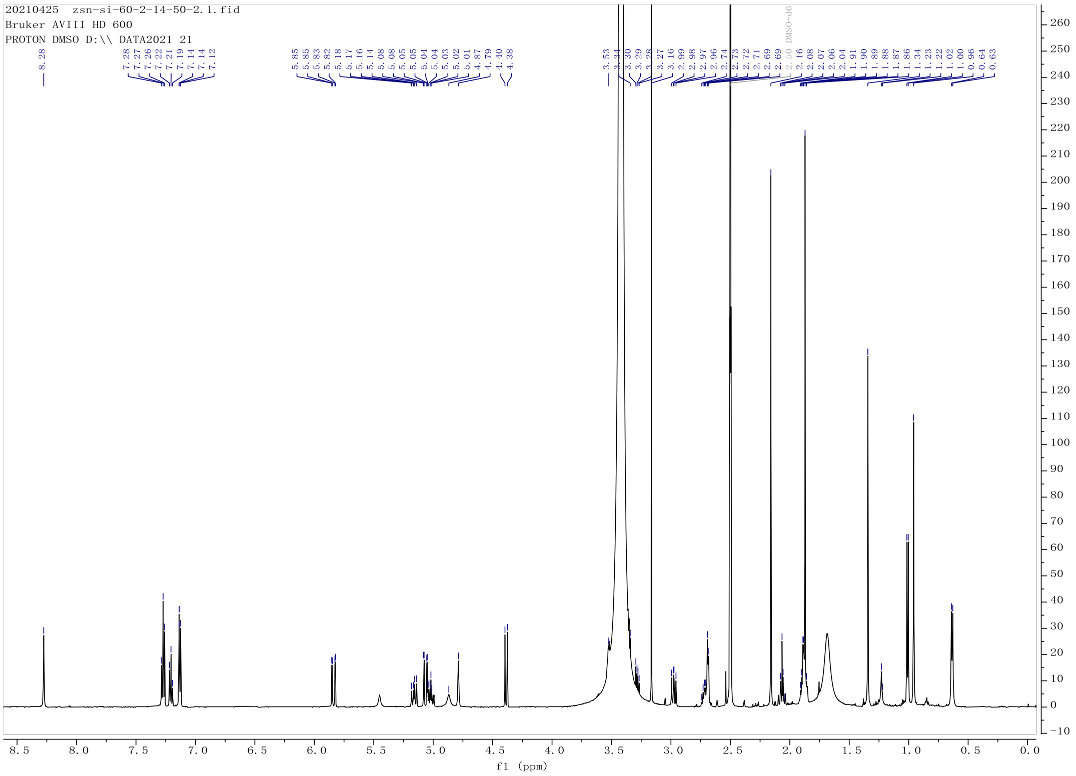

Figure S19: ^{13}C NMR (150 MHz, DMSO-d_6) spectrum of (**6**)

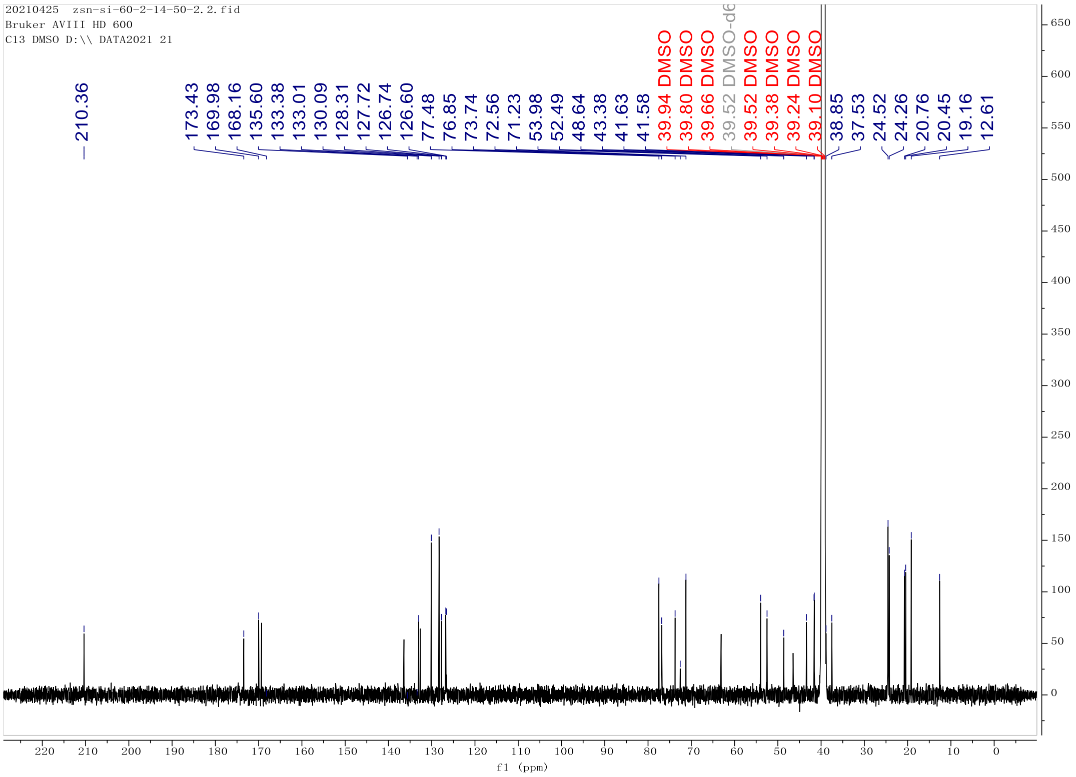

Figure S20: ^{1}H NMR (600 MHz, DMSO-d_6) spectrum of (**7**)

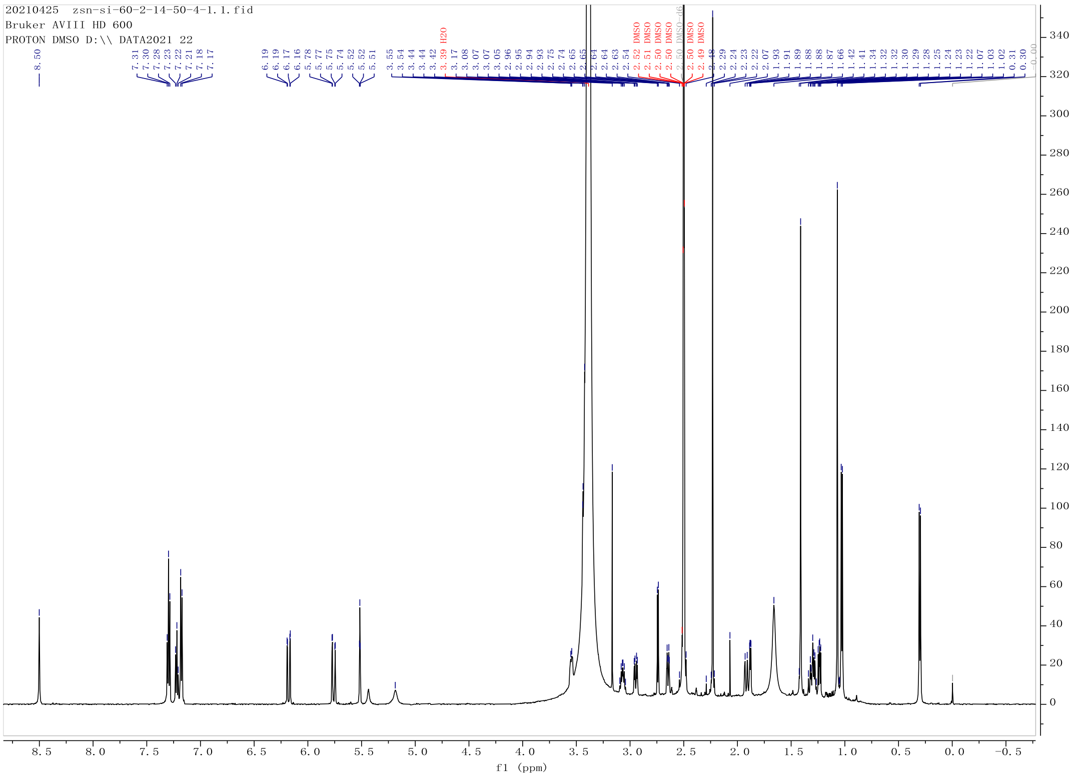

Figure S21: ^{13}C NMR (150 MHz, DMSO-d_6) spectrum of (**7**)

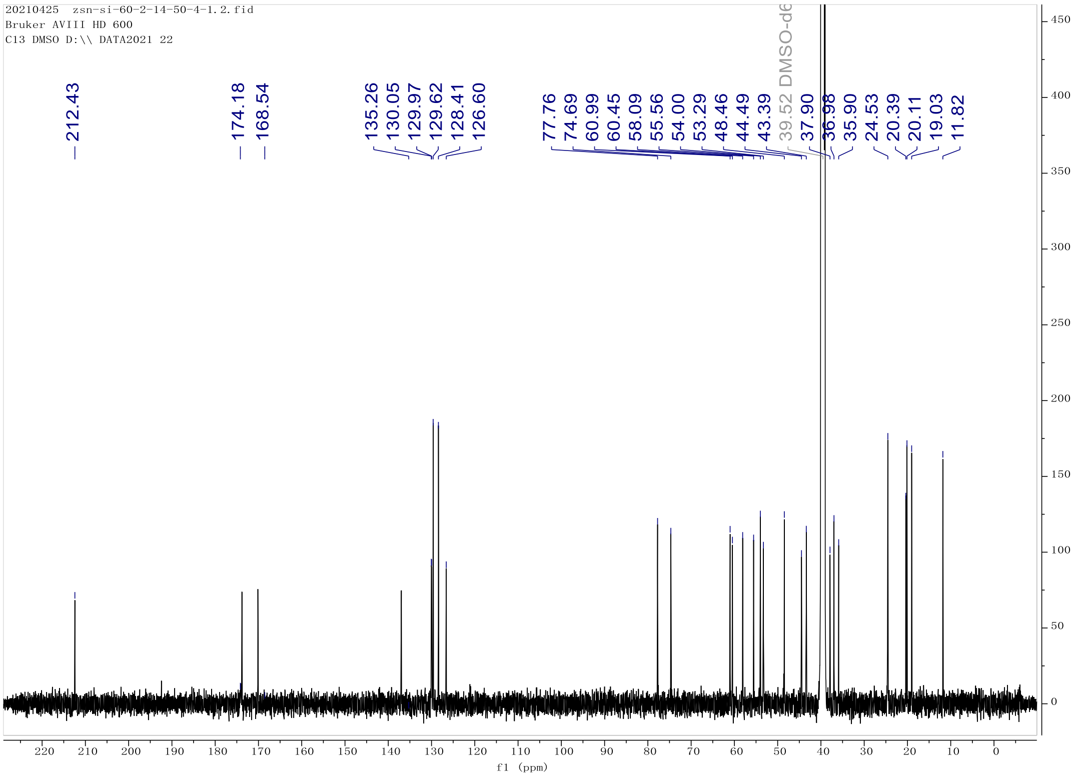

Figure S22: ^{1}H NMR (600 MHz, DMSO-d_6) spectrum of (**8**)

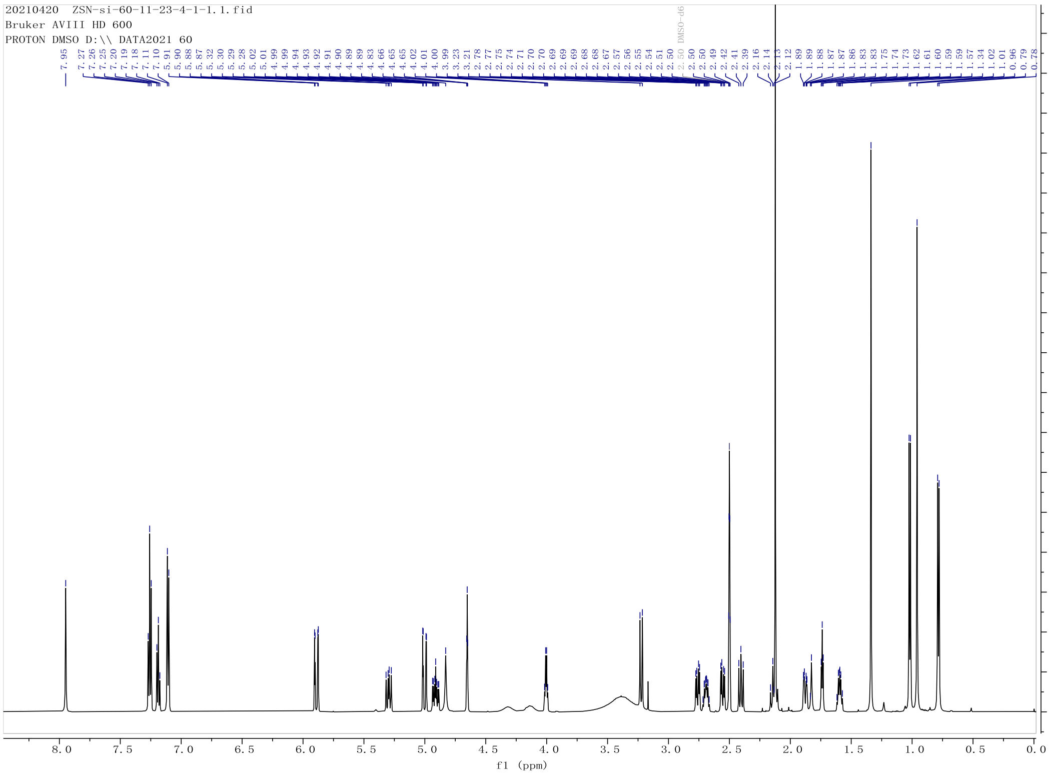

Figure S23: ^{13}C NMR (150 MHz, DMSO-d_6) spectrum of (**8**)

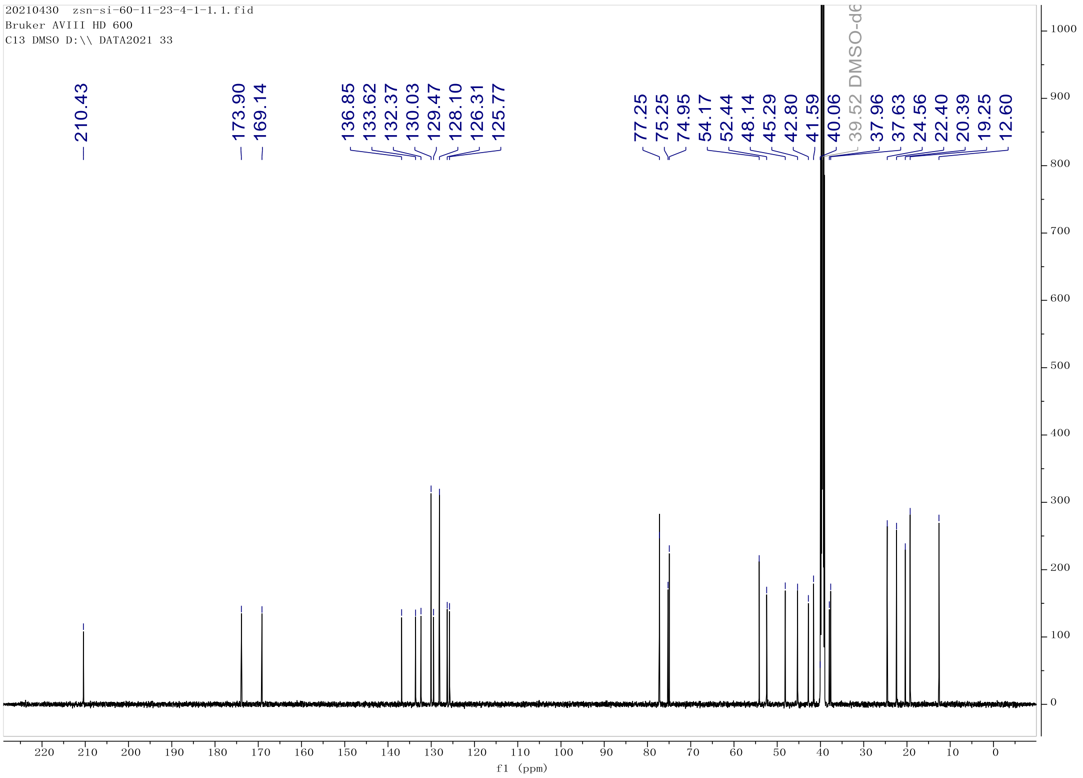

Figure S24: ^{1}H NMR (600 MHz, DMSO-d_6) spectrum of (**9**)

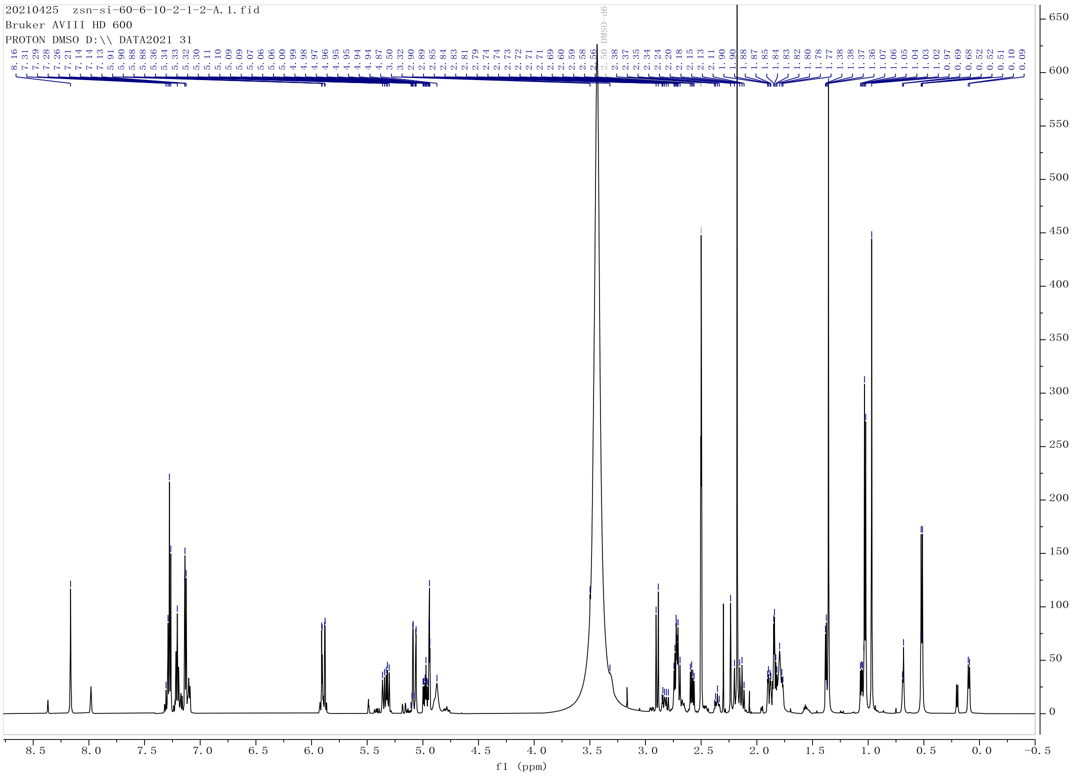

Figure S25: ^{13}C NMR (150 MHz, DMSO-d_6) spectrum of (**9**)

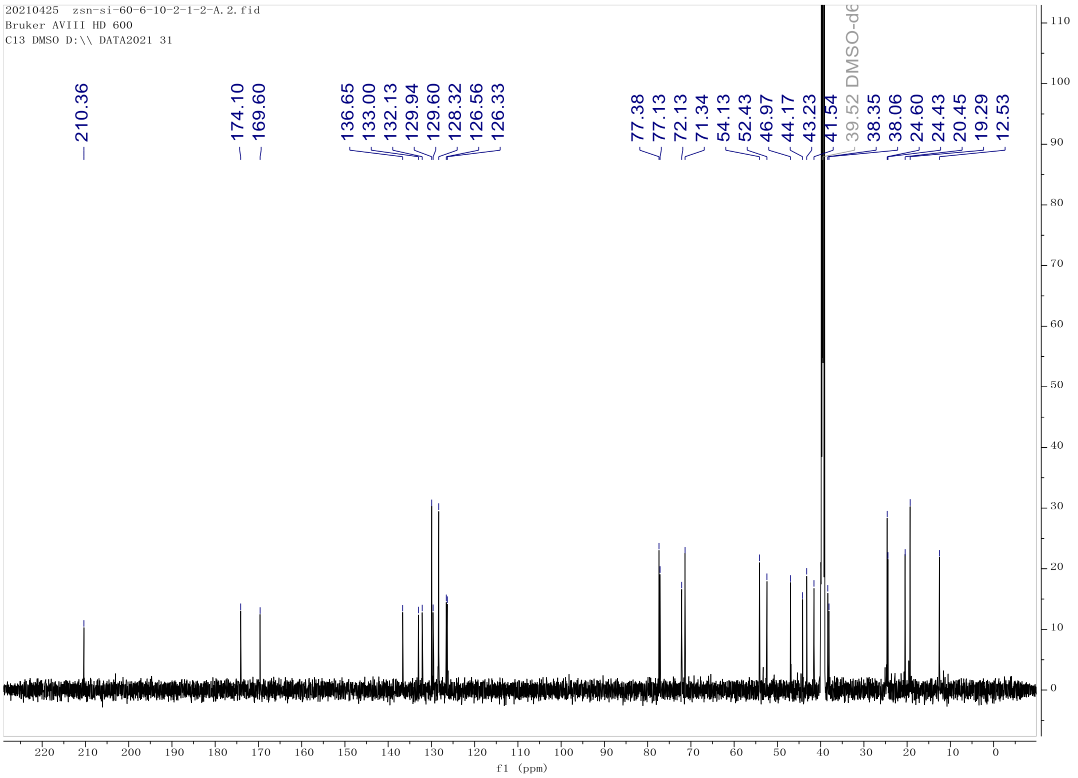

Figure S26: ^{1}H NMR (600 MHz, DMSO-d_6) spectrum of (**10**)

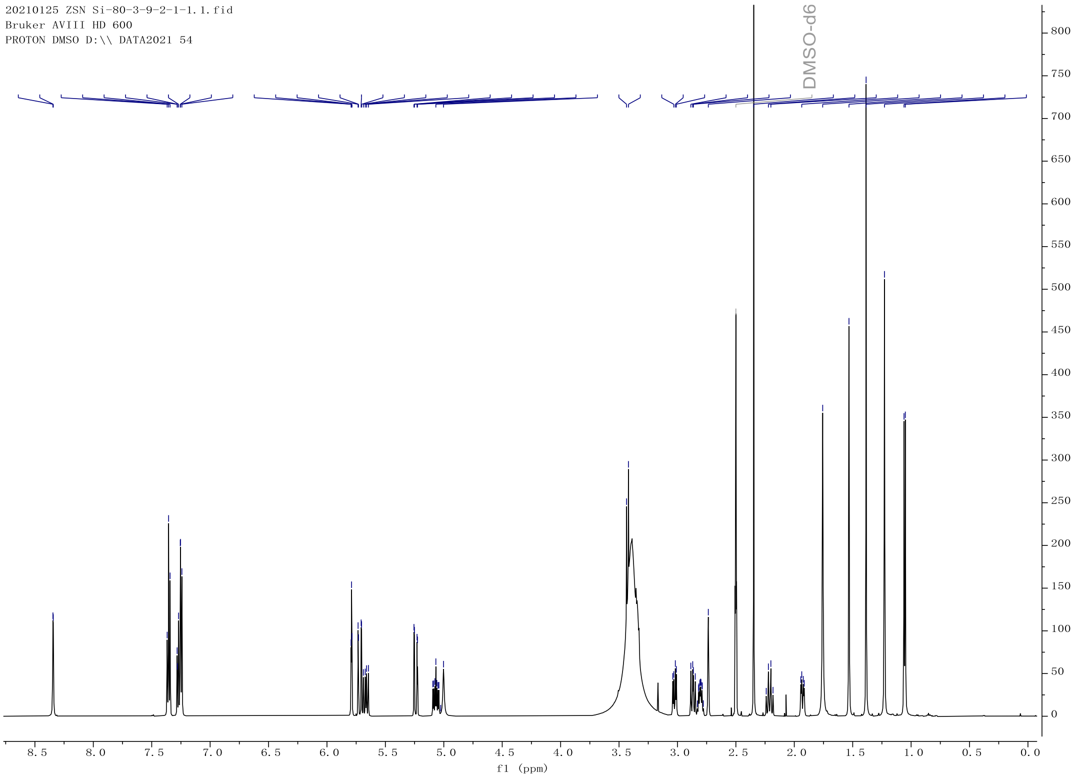

Figure S27: ^{13}C NMR (150 MHz, DMSO-d_6) spectrum of (**10**)

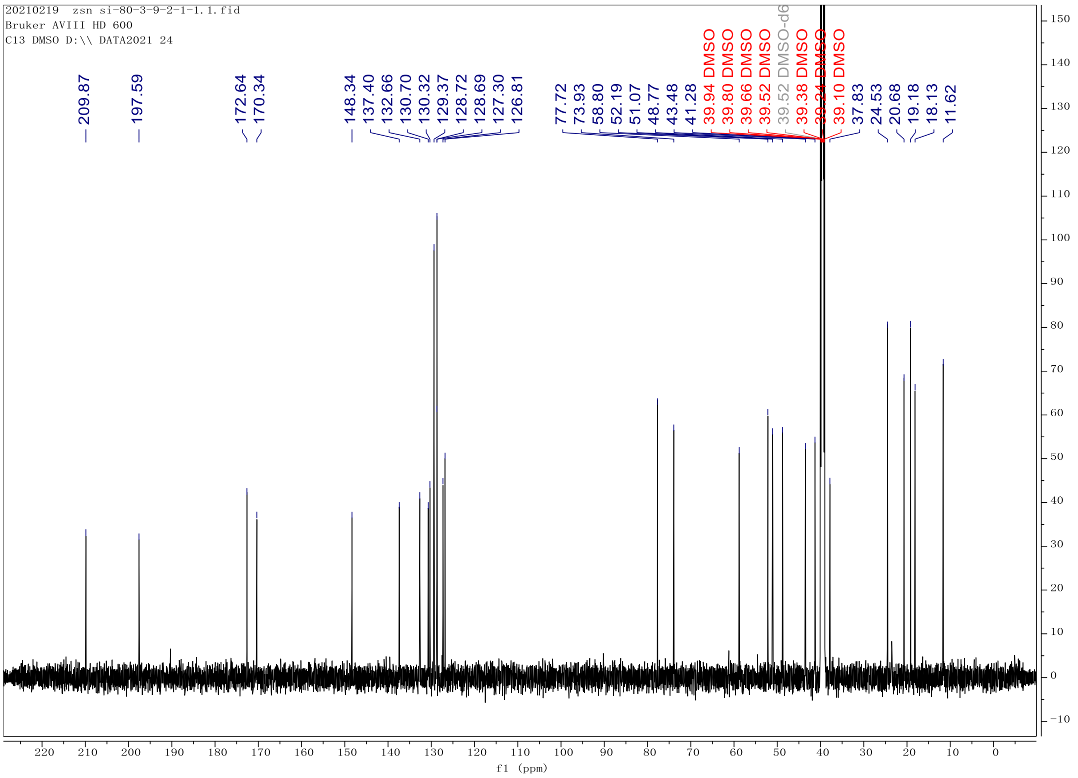

Figure S28: ^{1}H NMR (600 MHz, DMSO-d_6) spectrum of (**11**)

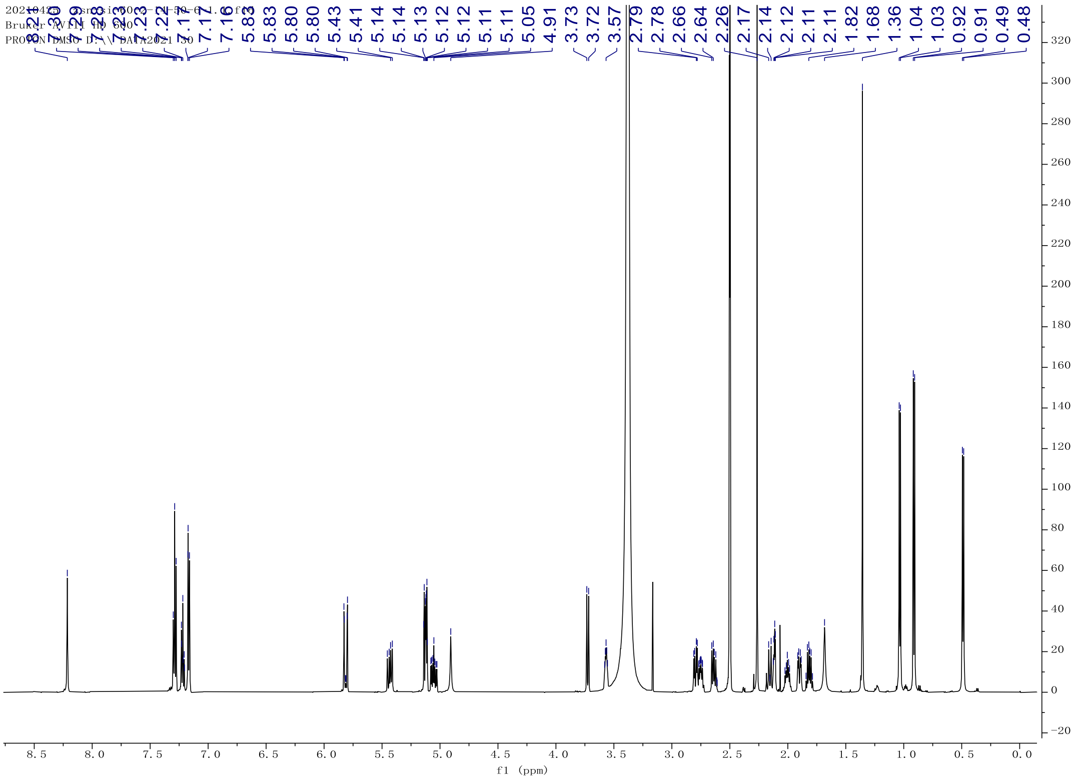

Figure S29: ^{13}C NMR (150 MHz, DMSO-d_6) spectrum of (**11**)

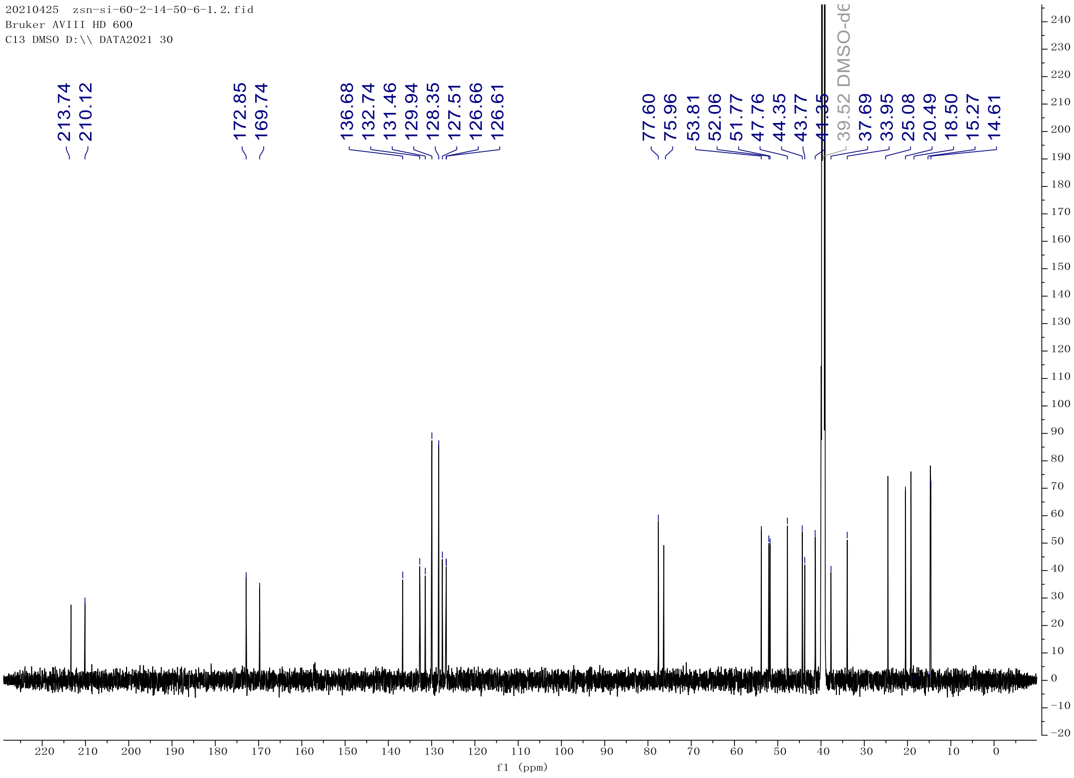

Supplement: Supplementary file 1 [file molecules-27-00136-s001.zip › molecules-1497740-supplementary.pdf]
